# The impact of diet quality on the velocity, morphology and normality of sperm in the zebra finch *Taeniopygia guttata*

**DOI:** 10.1242/jeb.243715

**Published:** 2022-05-09

**Authors:** Callum S. McDiarmid, Laura L. Hurley, Madiline Le Mesurier, Andrew C. Blunsden, Simon C. Griffith

**Affiliations:** Department of Biological Sciences, Macquarie University, Sydney, New South Wales 2109, Australia

**Keywords:** Sperm, Ejaculate, Condition dependence, Zebra finch, Sperm motility

## Abstract

Sperm traits can influence fertilisation success, but there is still much we do not understand about sperm condition dependence, that is, how much sperm traits depend on the male's energy acquisition and allocation. This is especially pronounced in avian taxa, despite extensive observational studies and sampling in wild populations. In this study, we collected sperm samples before and after experimentally reducing diet quality of wild-derived captive zebra finches in small mixed-sex groups, which we compared with individuals on a control diet. We measured the length of sperm components (head, midpiece, flagellum and total sperm length), the proportion of sperm with normal morphology, the proportion of sperm that were progressively motile and sperm swimming velocity (curvilinear velocity; VCL). The only sperm trait we found to be impacted by reduced diet quality was a significant decrease in sperm midpiece length. This is consistent with emerging evidence in other non-model systems, as well the fact that diet can alter mitochondrial density and structure in other tissue types. There was also a significant decrease in sperm velocity and the proportion of motile sperm over the course of the experiment for both experimental groups (i.e. unrelated to diet). This decrease in sperm velocity with largely unchanged sperm morphology emphasizes that there are other important determinants of sperm velocity, likely including seminal fluid composition.

## Abstract (precis)

**Summary:** A reduced diet quality in the zebra finch shortens the sperm midpiece but other sperm traits are unaffected.

## INTRODUCTION

Sperm quality can influence reproductive success in both competitive and non-competitive fertilisation scenarios (Snook, 2005; Fitzpatrick and Lüpold, 2014), making sperm variation across time, and environmental conditions, important for understanding fertilisation success and fitness. Ejaculates are metabolically costly to produce (Van Voorhies, 1992; Olsson et al., 1997), so ejaculate quality may be condition dependent, meaning that it may be impacted by the metabolic resources an individual can obtain and how they are allocated. The condition dependence of sperm traits is not necessarily evident from research into other aspects of sperm biology, as studies often rely on a single sperm sample per male collected under potentially varying environmental, social and dietary conditions (e.g. Simpson et al., 2014; Kleven et al., 2008; Rowe and Pruett-Jones, 2011; Lifjeld et al., 2010). Captive studies assessing individual repeatability do collect multiple samples, but often control external factors (e.g. diet, social context) to ensure the samples are comparable (e.g. Cramer et al., 2014; Opatová et al., 2015). In the wild, one could collect multiple sperm samples over time (e.g. Lüpold et al., 2012) and compare them to temporal changes in body mass index, but there may still be confounding factors influencing male condition (such as differences in territory quality amongst males). Sampling before and after experimental manipulations of nutrient availability therefore offers an opportunity to strengthen our understanding of intra-individual plasticity and the impact of nutrient availability on ejaculate traits (Macartney et al., 2019).

Fertilisation success can depend on multiple sperm traits (Simmons and Fitzpatrick, 2012; Lymbery et al., 2018). Sperm morphology may impact swimming velocity (Firman and Simmons, 2010; Bennison et al., 2015) and female sperm storage (Miller and Pitnick, 2002; Briskie and Montgomerie, 1992), both of which influence fertilisation success (Pizzari and Parker, 2009; Fitzpatrick and Lüpold, 2014). The proportion of sperm that develop abnormal morphology or are immotile is also important for fertilization success (Kruger et al., 1988; Rowe and Pruett-Jones, 2011), as are the various components of the non-sperm ejaculate, such as seminal proteins (e.g. Cornwallis and O'Conner, 2009; Perry et al., 2013). These different sperm traits can differ in their developmental trajectory, variation across time and among individuals, heritability and likely condition dependence (Macartney et al., 2019). The condition dependence of ejaculate traits also has implications for sperm competition theory (Simmons and Kotiaho, 2002; Wylde et al., 2020) and the existence of allocation trade-offs among ejaculate traits, or among ejaculate and life-history traits (e.g. Rahman et al., 2013; Ng et al., 2018; Magris et al., 2020).

A recent meta-analysis on the condition dependence of sperm traits found that with reduced nutrient intake there was a significant and ‘moderate’ decrease in ejaculate traits, but with notable heterogeneity across traits and taxa (Macartney et al., 2019). It also highlighted that there have been very few studies on condition dependence of bird sperm, despite the evolution and function of bird sperm traits receiving relatively considerable research attention (Macartney et al., 2019). The relatively limited number of studies on the condition dependence of avian sperm is probably because most avian studies have focused on birds in wild populations, whereas in other taxa, studies have more commonly focused on animals held in captivity. Macartney et al. (2019) also highlighted another gap in knowledge, which is that most studies have focused primarily on total sperm length, rather than examining the condition dependence of specific components of sperm morphology (head, midpiece and flagellum lengths).

The zebra finch (*Taeniopygia guttata* Reichenbach 1862) is one of the most widely researched avian species across many research areas (Griffith et al., 2021) including sperm biology (Birkhead, 2010). Zebra finch sperm morphology varies greatly among males, as in most estrildid finches (McCarthy et al., 2021), but within males, it is relatively consistent over time [repeatability (*R*)∼0.8; Opatová et al., 2015] with a strong genetic basis (Mossman et al., 2009; Knief et al., 2017; Kim et al., 2017). Interest in sperm morphology typically derives from understanding how it may impact sperm swimming velocity, which is reasonably well understood in the zebra finch, with midpiece length and quadratic flagellum length correlating with sperm velocity (Kim et al., 2017; Knief et al., 2017). There is emerging evidence that the sperm midpiece may be particularly susceptible to low condition or physiological stressors (Tomášek et al., 2017; Kahrl and Cox, 2015), which may be problematic as mitochondria in the sperm midpiece can provide energy for cellular functions and motility (Amaral et al., 2013). A previous study on the condition dependence of sperm traits in the zebra finch experimentally reduced condition by increasing foraging effort and requiring birds to fly 4.6 km every day (Birkhead et al., 1998). The study found that the group of birds with experimentally lowered condition had lower sperm velocity and sperm number, a comparable proportion of motile sperm and total sperm length to the control group, and surprisingly that the proportion of normal sperm was higher in low condition birds than it was in controls (Birkhead et al., 1998). These results raise some new questions, including: (1) whether specific sperm components are impacted by low condition, and (2) whether changes in specific sperm components are responsible for the observed decrease in sperm velocity in low condition birds (Birkhead et al., 1998). We believe that another study assessing the condition dependence of ejaculate traits in the zebra finch using some new approaches is worthwhile to replicate some parts of the earlier study (Birkhead et al., 1998) and address these new questions. Key methodological changes we can employ include measuring the length of specific sperm components (head, midpiece, flagellum), not just total sperm length, and non-destructive sperm sampling that allows sample collection before and after treatment (Girndt et al., 2017). In addition, by manipulating only food quality between experimental groups, we can also eliminate the potential for exercise to actually improve the health and condition of the birds (Birkhead et al., 1998).

In this study, we investigate how reduced diet quality impacted zebra finch sperm traits, including specific sperm morphological components. We experimentally reduced diet quality by feeding treatment birds low quality seed types and couscous. The control group received the standard mix of seed types fed to captive finches that provides an array of nutrients. We hypothesised that reduced diet quality would cause a decrease in: (1) the length of sperm morphological components (head, midpiece, flagellum and total length); (2) sperm swimming velocity, (3) the proportion of progressively motile sperm; and (4) the proportion of sperm with normal morphology. Bill colour, a well-established condition-dependent trait in the zebra finch (Birkhead et al., 1998; Bradbury and Blakey, 1998), was used to assess the efficacy of the treatment.

## MATERIALS AND METHODS

### Animals and experimental set-up

This study used wild-derived zebra finches that were 3–7 generations removed from individuals taken into captivity from the wild in arid north-western New South Wales in 2007 or 2010. All work was conducted according to national and international guidelines with approval of Macquarie University Animal Ethics Committee (Animal Research Authority 2017/054).

In total, 118 zebra finches (58 females and 60 males) were used in this experiment. A power analysis (using G*Power version 3.1.9.6) based on the impact of diet manipulation on sperm midpiece length in brown anoles (effect size *d*=1.3; Kahrl and Cox, 2015) suggested using a minimum of *N*=32 males, so our total of 60 males should give us adequate power to detect similar or slightly weaker effects. All of the birds were sexually mature and at least 6 months old, and were randomly distributed across aviaries with respect to age. Prior to this experiment, the birds had been held in two large aviaries, from which birds used here were moved into a row of 8 outdoor aviaries (4.1 m long×1.85 m wide×2.24 m high), each aviary holding 7–8 males and 7–8 females. In each new small aviary, half the individuals came from each of the two previous large aviaries. Bird IDs were allocated to aviary prior to physically moving the birds, so there was no opportunity for biasing birds to particular aviaries based on their apparent body condition or appearance. All 8 aviaries were kept on the usual high quality dry seed (Avigrain Finch Blue, Berkeley Vale, Australia) for an initial 2 weeks, after which the aviaries were assigned a diet. A coin was flipped for the diet of the first aviary, and then subsequent aviaries alternated between diet treatments. Groups were set up in early November, towards the end of austral spring. After the 2 week acclimation period, males were caught, weighed and their bill colour was measured by a spectrophotometer (see below) and sperm samples collected. After 30 days on experimental or control diets, all males were again caught and had mass, bill colour and sperm sampling repeated.

During the experimental period, the control diet continued receiving the usual dry seed diet (high quality diet; HQ) (Avigrain Finch Blue, Berkeley Vale, Australia), composed of equal parts White French Millet, Canary seed, Japanese Millet, Panicum and/or Red Panicum. The second group were shifted over 7 days to a reduced quality diet (low quality diet; LQ). The final LQ consisted of a mix of gatton panic (*Megathyrsus maximum*: 60%) and rye (*Lolium perenne*: 30%) seeds with uncooked couscous (10%). Examination of the remaining seed (and husks) showed that the birds primarily ate the gatton and couscous and did not attempt to eat the rye seed. During the treatment, LQ aviaries were given 100 g of the LQ diet in a 22 cm wide dish every morning for the 3 weeks following the 7 day transition. This time likely allows more than one complete full spermatogenic cycle during diet treatments; the duration of spermatogenesis is unknown in the zebra finch, but in non-passerine birds including the Japanese quail, domestic fowl and Barbary drake is 11–13 days (Lin and Jones, 1992; Jones & Lin 1993) and in the yellow-throated sparrow *Gymnoris xanthocolis* (a passerine), even slightly faster (Bhat and Maiti, 1988).

Bill colour was measured using spectral reflectance of the upper mandible and was taken in three consecutive scans from the centre of the bill using a USB2000+Miniature Fiber Optic spectrophotometer (Ocean Optics Inc., Dunedin, FL, USA), a xenon light source PX-2 (Ocean Optics Inc.) with a fibre-optic cable held at an angle of 90 deg to the bill and about 10 mm from the surface of the mandible with a plastic sheath that excluded ambient light. Reflectance data were captured using the AVASOFT 7 program (Avantes, Eerbeek, The Netherlands), and measurements were made by one person (S.C.G.). We used Pavo 2 (Maia et al., 2019) to obtain an average and smoothed reflectance spectra for each individual from the three replicates. We then normalised by maximum and minimum reflectance values. As zebra finch bills fade they lose saturation, but they also move from redder to more orange bills (hue), and we found that commonly used measures of saturation (S1R ‘Red saturation’; contribution of red spectral range to total brightness, S8; *R*_max_−*R*_min_/B2 mean brightness) and hue (H3; wavelength at *R*_mid_) were all tightly correlated, so for further analysis we used S1R. Colour analyses were performed by one person (C.S.M.) blind to the treatment groups that the individuals were assigned to.

### Sperm sample processing

Sperm were sampled via cloacal massage and sperm velocity was immediately measured using standard techniques (Rowe et al., 2015). Sperm samples were first diluted in a small amount of Dulbecco's Modified Eagle Medium (DMEM, Invitrogen Ltd) preheated to 40°C (the approximate body temperature of zebra finches; Calder, 1964; Hurley et al., 2018). The exact amount of DMEM depended on estimated quantity of sperm visible in the capillary, and ranged between 15 and 200 μl to obtain roughly comparable concentrations. A 6 μl aliquot was then loaded into a pre-heated slide chamber designed specifically to record sperm swimming speed in one field of focus (depth 20 mm; Leja, The Netherlands). We captured 5 s of six unique fields of view for each male (total 30 s per male), recorded at 400× magnification using a phase contrast microscope (XC41, Olympus, Japan) fitted with a heated stage plate (TP-S, Tokai Hit, Shizuoka, Japan) using a connected digital camera (Leegria HF G25, Canon, Japan).

Sperm videos were analysed using computer-assisted sperm analysis (CASA ImageJ; Wilson-Leedy and Ingermann, 2007) to quantify sperm velocity and the proportion of motile sperm. Sperm velocity can be influenced by a number of methodological aspects and so we used approaches consistent with the published literature (Cramer et al., 2019). Sperm were tracked for 1 s in each field of view (frame rate 25 frames s^−1^). We performed checks including the removal of non-sperm particles and cases where CASA software switched sperm mid-track, and control for the effects of drift using measures obtained from completely immotile sperm cells or non-sperm particles (McDiarmid et al., 2017; Hurley et al., 2018). Specifically, we considered sperm immotile if they had an average path velocity (VAP) below 30 μm s^−1^ or straight-line velocity (VSL) below 25 μm s^−1^, sperm tracked for less than 10 frames were excluded from velocity measurements. We excluded samples with fewer than 20 tracked sperm for analyses of the proportion of motile sperm. Samples with fewer than 20 motile sperm were excluded from VCL calculations, and sperm velocity measurements were performed by one person (C.S.M.) with files labelled by raw file numbers, meaning they were blind to male ID and treatment group.

With sperm tracks that passed these criteria, we quantified the proportion of motile sperm and calculated sperm swimming velocity as curvilinear velocity (VCL; see Rowe et al., 2015 for VCL justification). We performed analyses using both the full dataset and a subset of just the fastest 10% of sperm cells for each male.

Finally, for the samples used to assess sperm morphology and sperm normality, a small aliquot of the sperm was fixed in 5% buffered formaldehyde solution, and then used to create smears on glass microscope slides. For sperm normality we assessed 100 sperm each from two replicate smears as having either normal or abnormal sperm morphology (i.e. damage to entire sperm cell or visible abnormalities). These normality assessments were performed by one person (A.C.B.), while being blind to the treatment group samples belonged to. For sperm morphology, sperm were photographed using a phase contrast microscope (Olympus BX50, Olympus Japan) with a connected camera (14MP Aptima COMS, RisingCam). We photographed and measured the length of the head, midpiece and ‘tail’ (naked flagellum) and calculated flagellum length (midpiece+tail) and total sperm length (head+flagellum) for 10 morphologically normal sperm per male. The measurement of sperm morphology was performed using *Sperm Sizer* (McDiarmid et al., 2021), a freely available program that semi-automates the process of measuring sperm component lengths, and by one person (M.L.M.) while blind to the treatment group samples belonged to.

### Statistical methods

The statistical analyses were conducted in R version 4.1.2 (https://www.r-project.org/) and used RStudio version 1.2.5033 (https://www.rstudio.com/) as the graphical user interface.

We used linear mixed-effect models in the lme4 package (Bates et al., 2015; https://CRAN.R-project.org/package=lme4) to test whether diet manipulation impacted sperm traits, bill colour and body mass. These sperm traits were sperm swimming velocity (VCL) including either all sperm in a sample or only including the fastest 10% of sperm in the sample, the proportion of normal sperm (logit transformed), the proportion of progressively motile sperm (logit transformed), and the length of sperm components (head, midpiece, flagellum and total sperm length). Model assumptions were checked by examining residuals. The dependent variable of each model had the sperm component of interest, bill colour or body mass. The fixed effects were time point (before or after treatment) and experimental group (LQ or HQ) and their interaction. The random effects always included aviary ID and male ID, and also included sample ID for measures with multiple datapoints per sample (i.e. velocity and morphology measures) and field of view ID for velocity models. Non-significant interaction terms were removed to give the final model. All models assumed a Gaussian distribution, and we logit transformed the proportion of motile sperm and the proportion of normal sperm.

For these models we calculated marginal *R*^2^ to show the amount of variance explained by fixed factors, the intra-class correlation coefficients (ICCs) to show the variance explained by each random effect, and the conditional *R*^2^ to show the total variance explained by the model (Nakagawa and Schielzeth, 2010, 2012).

We also tested for a relationship between sperm swimming velocity and sperm component length, as has been found in other captive populations of zebra finch (Bennison et al., 2015; Knief et al., 2017; Kim et al., 2017). We tested this separately for both the samples collected before treatment, the samples collected after treatment, and all samples together. We followed the methodology of Knief et al. (2017). We fitted a linear model (lme4 package; Bates et al., 2015) with explanatory variables as mean-centred head, midpiece and flagellum lengths (averaged per male), all two-way interactions between them, and their squared terms. We then performed stepwise removal of the least significant terms until only significant terms remained. We also performed this analysis for the average VCL of the fastest 10% of sperm per sample. When considering the combined datasets (data from both time points) male ID was initially included as a random effect (making a linear mixed model), but was removed as it explained virtually zero variation.

*Post hoc* we tested whether, among males, the change in sperm midpiece length correlated to change in VCL, or change in bill colour saturation, using a linear model.

## RESULTS

**Fig. 1.**
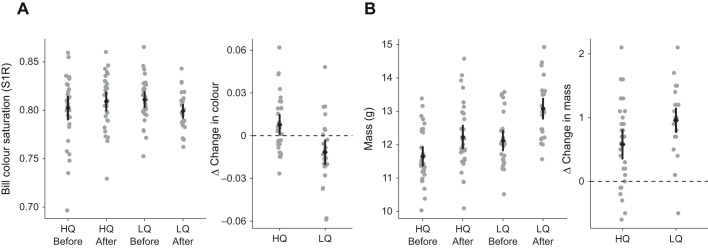
**Bill colouration and body mass of male zebra finches before and after diet manipulation (high quality diet HQ, or low quality diet LQ), and the change between these two time points (calculated per-individual).** Raw data points (one per male) are shown in grey, and the mean and 95% confidence interval of the mean are shown in black for (A) bill colour and (B) body mass. Samples sizes for both bill colour and mass were: HQ before, *n*=31; HQ after, *n*=30; LQ before, *n*=29; LQ after, *n*=26. Data were analysed with linear mixed models. Bill colour saturation showed a significant interaction between sampling time point and diet treatment group (*P*=0.0013; Table 1), increasing over time for the HQ group and decreasing for the LQ group. Body mass also showed a significant interaction between sampling time point and diet treatment group (*P*=0.0217; Table 1), with both groups increasing over the treatment, with LQ having significantly higher mass than HQ after treatment. The experiment was not replicated in the laboratory.

Bill colour saturation showed a significant interaction between the sampling time point (before or after treatment) and diet treatment group (HQ or LQ), with bill colour saturation increasing over time for the HQ group and decreasing for the LQ group (Table 1; Fig. 1A). Body mass also showed a significant interaction between time point and diet group and both groups increased in mass over the experiment, with LQ birds having significantly higher mass than HQ birds after treatment (Table 1; Fig. 1B).

**Table 1.**
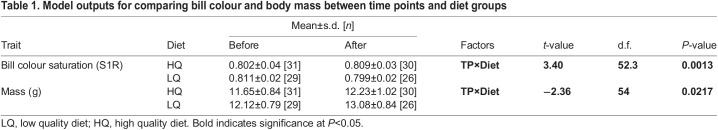
Model outputs for comparing bill colour and body mass between time points and diet groups

In some cases, it was not possible to collect sperm. In other cases, enough sperm could be collected to obtain an estimate of the proportion motile but not enough could be found and photographed in the fixative to measure sperm morphology. Of the 31 males that started in the HQ group, 30 survived, of which all could be sampled for proportion motile before and after treatment, 27 for sperm morphology before and 29 after treatment. Of the 29 males that started in the LQ group 26 males survived, 29 could be sampled for the proportion of motile sperm before and 20 after treatment, 22 could be sampled for morphology before and 17 after treatment (Table 2). A chi-squared test found no difference between treatment groups or time point in the likelihood that sperm could be sampled for either proportion motile (χ^2^=0.91, d.f.=1, *P*=0.34) or for sperm morphology (χ^2^=0.28, d.f.=1, *P*=0.60).

**Table 2.**
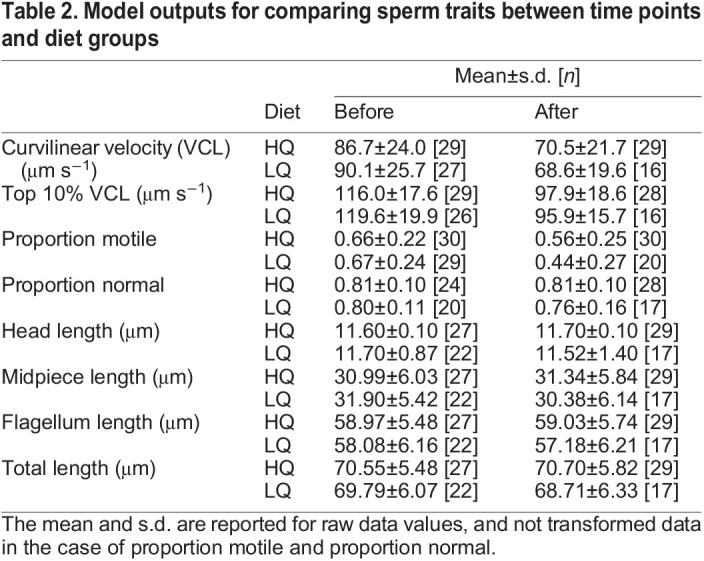
Model outputs for comparing sperm traits between time points and diet groups

**Fig. 2.**
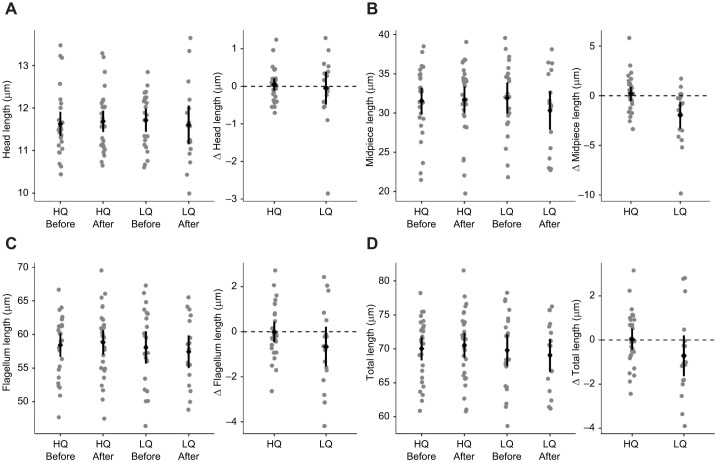
**Length of sperm components including head, midpiece, flagellum and total sperm length before and after the diet manipulation, and the change between these two time points.** Raw data points (one per male) are shown in grey, and the mean and 95% confidence interval of the mean are shown in black for (A) head, (B) midpiece, (C) flagellum and (D) total sperm length. Samples sizes for both bill colour and mass were: HQ before, *n*=27; HQ after, *n*=29; LQ before, *n*=22; LQ after, *n*=17. Data were analysed with linear mixed models. Sperm midpiece was the only sperm component that had a significant result, with a significant interaction between time point and diet (*P*=0.006; Table 3). The experiment was not replicated in the laboratory.

There was no significant effect of time point, diet or their interaction on head, flagellum or total sperm length (Table 3; Fig. 2A,C,D). There was a significant time point and diet interaction for sperm midpiece length, with the LQ group midpiece shortening over the course of the treatment, and the HQ group showing no change (Table 3; Fig. 2B). For the LQ males, change in midpiece length did not correlate with change in VCL (*t*-value=0.87, d.f.=13, *P*=0.40) or bill colour saturation (*t*-value=1.08, d.f.=13, *P*=0.30).

**Table 3.**
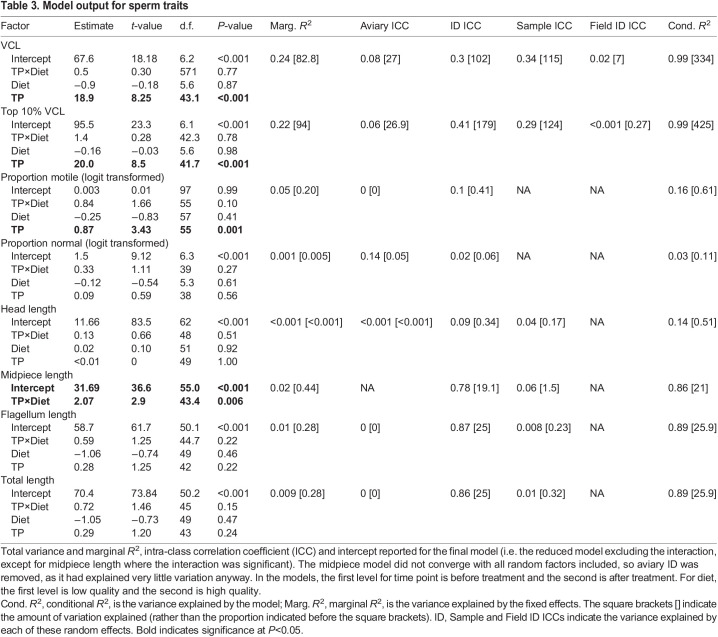
Model output for sperm traits

**Fig. 3.**
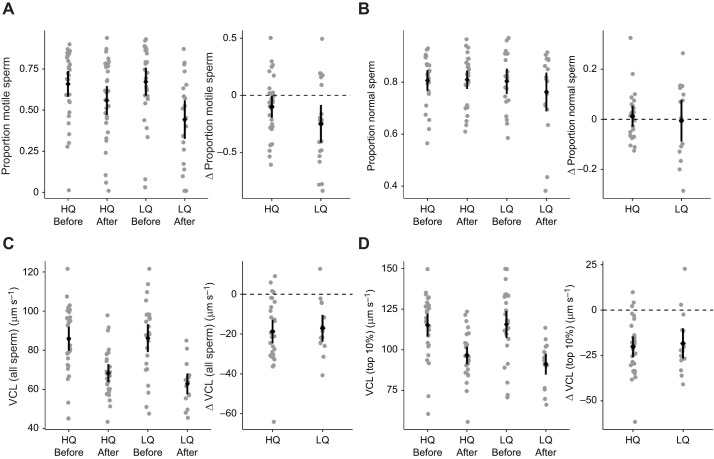
**The proportion of motile sperm, the proportion of normal sperm and the curvilinear velocity (VCL) averaged either for all sperm in a sample or just the fastest 10% of sperm in a sample.** (A) Proportion of motile sperm, (B) proportion of normal sperm, (C) VCL for all sperm and (D) VCL for fastest 10% are presented before and after the diet manipulation, and the change between these two time points, grouped by experimental treatment group (high quality diet HQ, or low quality diet LQ). Raw data points (per male) are shown in grey, and the mean and 95% confidence interval of the mean are shown in black. Samples sizes for VCL were: HQ before, *n*=29; HQ after, *n*=29; LQ before, *n*=27; LQ after, *n*=16; for top 10% VCL: HQ before, *n*=29; HQ after, *n*=28; LQ before, *n*=26; LQ after, *n*=16; for proportion motile: HQ before, *n*=30; HQ after, *n*=30; LQ before, *n*=29; LQ after, *n*=20; proportion normal: HQ before, *n*=24; HQ after, *n*=28; LQ before, *n*=20; LQ after, *n*=17. Data were analysed with linear mixed models. The proportion of normal sperm did not change for either diet group (*P*>0.05), but there was a significant effect of time point for the proportion of motile sperm (*P*=0.001), the VCL when averaged for all sperm in a sample (*P*<0.001) and the VCL of the fastest 10% of sperm per sample (*P*<0.001; Table 3). The experiment was not replicated in the laboratory.

The proportion of normal sperm did not change for either diet group (Table 3; Fig. 3B). However, there was a significant decrease over the course of the experiment for all measures of sperm motility; the proportion of motile sperm, the VCL when averaged for all sperm in a sample, and the VCL of the fastest 10% of sperm per sample (Table 3; Fig. 3A,C,D).

Regarding the relationship between sperm morphology and sperm velocity, we found that when considering samples collected before the diet treatment, the model that best explained average VCL was a significant quadratic relationship with the average flagellum length (Table S1). When considering samples from after the diet treatment, sperm midpiece and flagellum length significantly predicted average VCL (Table S1). Combining data from both time points resulted in a significant quadratic relationship between VCL and average flagellum length (Table S1). Considering only the fastest 10% of sperm per sample gave the same results, except that when only considering data from after the diet treatment there was no significant relationship between any morphological measures and VCL (Table S1).

## DISCUSSION

Ejaculate traits can be key to male reproductive success and are energetically expensive to produce, so realised trait expression may depend on the male's energy acquisition and allocation. In the present study, we found heterogeneous changes across sperm traits that further our understanding of the condition dependence and plasticity of these traits. The diet treatment significantly impacted bill colouration, with an increase in bill colour saturation for HQ males, and a decrease for LQ males, indicating the efficacy of our dietary treatment. Sperm midpiece length significantly shortened for LQ males, but showed no difference for HQ males, consistent with other emerging evidence that sperm midpiece can be condition dependent (e.g. Bonanno and Schulte-Hostedde, 2009; Kahrl and Cox, 2015; Ros-Santaella et al., 2019; but not Devigili et al., 2013). While the length of other sperm components and the proportion of normal sperm did not significantly change over the course of the experiment for either treatment group, there was a significant decrease in sperm velocity and the proportion of motile sperm over the course of the experiment in both treatment groups. This suggests that the slowing of sperm was mediated by something other than variation in sperm morphology. We discuss each of these results in turn below.

Sperm midpiece length was the only morphological component impacted by the diet manipulation (Fig. 2). There is emerging evidence that sperm midpiece size is impacted by manipulation of diet quality or related to some measure of condition, although in some cases the relationship is negative (brown anole, Kahrl and Cox, 2015; red squirrel, Bonanno and Schulte-Hostedde, 2009) and in some it is positive (fallow deer, Ros-Santaella et al., 2019; zebra finch, Tomášek et al., 2017; present study). In the present study, midpiece shortening for the LQ group may mean there is less mitochondrial material in these sperm (Knief et al., 2019; but see Mendonca et al., 2018), potentially reducing ATP availability (Rowe et al., 2013; but see Bennison et al., 2016). Mitochondria in other cell types are known to regulate their density, size and activity in response to cellular conditions (Rambold et al., 2011; Liao et al., 2016; Alcántar-Fernández et al., 2019; Bañuls et al., 2019), including reducing mitochondrial quantity in response to malnutrition (Park et al., 2003; Zhang et al., 2020; Fan et al., 2021). Closely regulating mitochondria depending on nutrient availability and energy demand may be important to minimise oxidative stress, which sperm are particularly at risk of (Tomášek et al., 2017; Helfenstein et al., 2010; Friesen et al., 2019, 2020). A recent meta-analysis (Macartney et al., 2019) could not assess the condition dependence of particular sperm components (i.e. head, midpiece and flagellum) as too few studies had reported them, but did find that total sperm length had a small, negative response to nutrient limitation (Macartney et al., 2019). Data from our experiment emphasises the overall stability of sperm morphology for each zebra finch male, with no significant change for most components (head, flagellum and total length). This is consistent with previous work reporting high repeatability of sperm morphology in the zebra finch (Opatová et al., 2015), and a previous study that found no condition dependence of total sperm length in the zebra finch (Birkhead et al., 1998).

Sperm velocity and the proportion of motile sperm decreased over the course of this experiment for males in both groups, and the cause is unknown (Fig. 3). It is unlikely to be due to seasonal effects as this experiment was performed during the austral spring and summer, and in any case zebra finches are opportunistic breeders that can breed at any time of year, even in poor conditions (Griffith et al., 2021). It is also unlikely to be due to ageing, as 30 days is a relatively short time compared with the typical lifespan of several years for zebra finches in captivity (S.C.G., personal observation). Some previous studies have also not found a clear response of zebra finch sperm velocity to other stressors, including oxidative stress (Tomášek et al., 2017) or simulated heatwaves (Hurley et al., 2018). Intriguingly, there is also evidence that sperm velocity may decrease under desirable breeding conditions, with one study showing that velocity of the fastest 10% of sperm decreased across successive clutches when zebra finch pairs successfully fledged nestlings, but did not decrease across clutches for unsuccessful pairs (Hurley et al., 2020). However, it is also possible that the dramatic decrease in sperm velocity across both groups owing to an unknown factor overwhelmed a difference between experimental groups. This would be more consistent with a previous study on condition dependence of zebra finch sperm (Birkhead et al., 1998), in which sperm velocity decreased in response to the condition manipulation. Meta-analytic evidence across taxa found that sperm motility showed a small, negative response to nutrient limitation (Macartney et al., 2019). While we found that flagellum and midpiece length did predict sperm swimming velocity to some degree (Table S3), the major change in sperm velocity between time-points that we observed in the absence of change in morphology (in the HQ group) highlights that there are important factors other than sperm morphology impacting sperm velocity. This emphasises that researchers should look beyond sperm morphology to understand the basis of sperm velocity in passerine birds, for example the non-sperm ejaculate (Cornwallis and O'Conner, 2009).

In conclusion, a low-quality diet significantly reduced sperm midpiece length, but did not impact the length of other sperm components (head, flagellum, total) or the proportion of normal sperm. The significant shortening in sperm midpiece is consistent with emerging evidence of other taxa, and the known plasticity of mitochondrial structure and function in other cell types in response to altered diet (Zhang et al., 2020; Fan et al., 2021). We found that sperm velocity and proportion of motile sperm did not differ between dietary treatment groups but significantly decreased over the course of the experiment (in both experimental and control birds) by roughly 25%. This highlights that we poorly understand the determinants of sperm velocity, that different factors impact sperm morphology and velocity, and experimentally confirms that sperm morphology is typically consistent for male zebra finches over time. This work emphasises the value of assessing the condition dependence of specific sperm components (e.g. midpiece length) rather than just total sperm length, particularly in taxa where there are known fertility implications (e.g. midpiece in house mice and deer mice, Firman and Simmons, 2010; Fisher et al., 2016). Future research is needed on the determinants of sperm velocity as an extremely plastic trait, which will likely mean measuring and perhaps manipulating components of the sperm environment, including non-sperm components of the ejaculate (Reinhardt et al., 2015).

## Supplementary Material

Supplementary information
