# Supplementary material for: The impact of diet quality on the velocity, morphology and normality of sperm in the zebra finch Taeniopygia guttata
Source: J Exp Biol. 2022 May 9;225(9):jeb243715. doi: 10.1242/jeb.243715 (PMC9163447; doi:10.1242/jeb.243715)
Supplement: Supplementary information [file jexbio-225-243715-s1.pdf]

Table S1. Output from the linear models of sperm morphology and sperm velocity. For sperm velocity we used either the average curvilinear velocity (‘VCL’) per sample, or the VCL of the fastest 10% of sperm in a sample (‘Top 10% VCL’). Measures of head, midpiece and flagellum lengths were averaged per sample and mean centred. We performed the models for with data before and after the experimental treatment, and both datasets combined. For each model we performed a stepwise removal of all non-significant terms, terms are listed in order of removal. Note that VCL and Top 10% VCL are highly correlated. R^2 refers to multiple R-squared. Intercept included for full model. For the combined datasets, male ID was initially included as a random factor, but removed as it explained basically zero variation (ICC = 0).

Click here to download Table S1

Journal of Experimental Biology • Supplementary information
